# Awareness of Heart Attack Symptoms and Risk Factors Among the Elderly in Rural Chennai: A Cross-Sectional Study

**DOI:** 10.7759/cureus.85692

**Published:** 2025-06-10

**Authors:** Sahasyaa Adalarasan, Kanika Balasubramani, Nithyashree Sathiyamurthy, Vedasree Karthikeyan, Sudharshini Subramaniam

**Affiliations:** 1 Institute of Community Medicine, Madras Medical College, Chennai, IND

**Keywords:** awareness, heart attack, myocardial infarction, risk factors, rural, symptoms

## Abstract

Aims and objectives

Myocardial infarction (MI) remains a leading cause of mortality and morbidity globally. Timely recognition of symptoms, and understanding of associated risk factors, are critical for early intervention and improved outcomes. However, awareness levels - particularly among the elderly in rural regions - remain poorly studied. This study aimed to assess the awareness of heart attack symptoms and risk factors among the elderly population in rural Chennai.

Methodology

A cross-sectional study was conducted among 384 individuals, aged 60 years and above, in the rural field practice area of Medavakkam, under Madras Medical College, Chennai, India. An interviewer-administered questionnaire assessed knowledge of 9 symptoms and 11 risk factors associated with MI. Awareness scores were calculated based on participants’ spontaneous and prompted responses.

Results

The mean age of participants was 65 years. Chest pain (248, or 64.58%) and breathlessness (176, or 45.83%) were the most recognized symptoms, while neck pain (12, or 3.12%) was the least known. Among risk factors, hypertension (182, or 47.4%) and stress (178, or 46.35%) were most identified, whereas high salt intake (22, or 5.73%) and obesity (26, or 6.77%) were least known. The average awareness score for symptoms was 5.56, for risk factors, 7.61, and the total knowledge score was 13.18 out of 20.

Conclusion

The study reveals significant gaps in awareness of heart attack symptoms and risk factors among the rural elderly. These findings underscore the urgent need for community-based health education and preventive strategies, tailored to this vulnerable demographic, to enhance early recognition and reduce MI-related morbidity and mortality.

## Introduction

Myocardial infarction (MI), pharmacologically defined as the irreversible death of heart muscle due to prolonged ischemia from coronary artery blockage, is a leading global cause of death. Sources report 17.9 million cardiovascular deaths annually, with 85% due to heart attacks and strokes [1]. The seriousness of MI lies in its ability to cause rapid deterioration of heart function, as the dead tissue is unable to function properly. This often leads to death or permanent disability if not treated in time. It is a life-or-death situation, where timely detection and intervention can make all the difference in survival rates. The "golden time" period, referring to the critical window of time after the onset of symptoms, is crucial for determining the outcome of a heart attack [2]. Early recognition of symptoms - such as chest pain, shortness of breath, dizziness, or nausea - along with prompt medical intervention, could significantly increase the chances of survival and reduce the risk of long-term complications [3].

The risk factors for MI include hypertension, diabetes, high cholesterol levels, smoking, physical inactivity, obesity, and a family history of heart disease [4]. The elderly, particularly those over 60, are at a heightened risk due to the cumulative effect of these risk factors over the years. Awareness of these symptoms and risk factors can, therefore, be a crucial determinant in the prevention and early detection of heart attacks.

This study aims to assess the level of awareness about heart attack symptoms and risk factors among the elderly population in rural Chennai, with a focus on communities like Medavakkam. Although Medavakkam is officially part of the Chennai Metropolitan Area, certain pockets still exhibit rural characteristics in terms of healthcare access and literacy. In Tamil Nadu, where less than 10% of the population is estimated to receive timely intervention, the findings of this study are particularly significant [5]. By identifying gaps in knowledge, the study emphasizes the need for focused education and preventive measures tailored to this vulnerable group. The results have important implications for healthcare policies and public health programs, offering insights that can help reduce heart attack-related deaths and improve health outcomes in rural communities.

## Materials and methods

Study design

This was a community-based, cross-sectional study conducted from April 5th to April 26th, 2025, among elderly individuals aged 60 years and above, residing in the rural field practice area of Medavakkam, affiliated with Madras Medical College (MMC), Chennai, India. Medavakkam is a semi-urban locality with an approximate population of 22,000, comprising mostly middle- to lower-middle-income households. The area has a literacy rate of around 82% and moderate access to primary healthcare services.

Ethical approval

Prior to data collection, ethical approval was obtained from the Institutional Ethics Committee (IEC) of MMC on April 4th, 2025 (approval no. 61042025). Written informed consent was obtained from all participants after explaining the study’s purpose, procedures, voluntary nature, and assurance of confidentiality.

Participant criteria

Participants included elderly individuals aged 60 years or above, who were permanent residents of the Medavakkam rural field practice area, and who were willing and able to provide informed consent. The number 60 comes from the WHO definition of elderly individuals [6]. Individuals who were debilitated or unable to comprehend the questionnaire were excluded from the study.

Sample size calculation

The sample size was calculated using the standard formula for estimating proportions, assuming maximum variance (p = 0.5), a 95% confidence level (Z = 1.96), and a 5% margin of error (d = 0.05). Based on these parameters, the minimum required sample size was 384.

Sampling method

Systematic random sampling was used to select participants from a sampling frame of elderly individuals registered in the Medavakkam Rural Health Training Centre database. Every nth eligible individual was approached until the desired sample size was achieved.

Data collection

Data were collected using an interviewer-administered questionnaire (see Appendices, Table 5), adapted from previously validated instruments used in similar studies [7]. The questionnaire was translated into the regional language (Tamil) and back-translated to English to ensure semantic and content equivalence. A pilot test was conducted on a small group of 10 individuals (excluded from final analysis), and the tool was reviewed by subject experts for face validity. The questionnaire comprised two sections: one assessing awareness of risk factors, and the other assessing knowledge of symptoms related to the condition. During the interview, participants were first asked to freely list any symptoms or risk factors they were already aware of, which were then checked off in the questionnaire. The interviewer subsequently asked about the remaining items. Each correctly identified item was awarded a score of 1, while unrecognized items were scored 0. The risk factor awareness score ranged from 0 to 11, the symptom awareness score from 0 to 9, and the total knowledge score from 0 to 20, with higher scores indicating greater awareness.

Data analysis

Data were analyzed using IBM SPSS Statistics for Windows, Version 24 (Released 2016; IBM Corp., Armonk, NY, USA). Descriptive statistics were used to summarize demographic characteristics and awareness levels. The prevalence of awareness was calculated using proportions and presented as frequencies and percentages. Mean scores were computed for both risk factors and symptom awareness and reported with standard deviations.

## Results

A total of 384 people participated in the present study. The demographic data of the study population are presented in Table 1. The mean age of the participants was found to be 65 years. Nearly equal participation from both genders was observed, with 184 (47.92%) females enrolled in the study. Regarding educational status, 323 (84.11%) participants were found to be elementary school graduates. In terms of personal and family medical history, 23 (5.9%) and 46 (10.68%) participants reported having had an MI themselves and having had a family member with an MI, respectively. With respect to comorbid conditions, 90 (23.44%) participants reported having diabetes mellitus and hypertension.

**Table 1:** Demographic characteristics of the study population MI, myocardial infarction; DM, diabetes mellitus; HTN, hypertension

Demographic characteristic	Prevalence (n = 384)
Age (mean, range)	65 (60-83)
Female gender	184 (47.92%)
Elementary school graduates	323 (84.11%)
High school graduates	41 (10.68%)
College graduates	20 (5.21%)
History of MI	23 (5.9%)
Family history of MI	42 (10.94%)
Presence of other comorbidities like DM/HTN	90 (23.44%)

The prevalence of knowledge of symptoms is summarized in Table 2. Among the symptoms, chest pain was the best known, with 248 (64.58%) participants being aware of it. Other appreciably well-known symptoms among the participants were breathlessness (176 participants, or 45.83%), light-headedness (176 participants, or 45.83%), and shoulder pain (164 participants, or 42.71%). The symptoms with the poorest awareness among participants were epigastric pain (68 participants, or 17.71%), nausea (64 participants, or 16.67%), back pain (44 participants, or 11.46%), and neck pain (12 participants, or 3.12%).

**Table 2:** Prevelance of knowledge of symptoms among the study population

Heart attack symptoms	Prevalence of knowledge
Chest pain	248 (64.58%)
Breathlessness	176 (45.83%)
Light-headedness	176 (45.83%)
Pain in shoulder/arm	164 (42.71%)
Epigastric pain	68 (17.71%)
Nausea	64 (16.67%)
Cold sweat	116 (30.21%)
Back pain	44 (11.46%)
Neck pain	12 (3.12%)

The prevalence of knowledge of risk factors is summarized in Table 3. Among the risk factors, hypertension and stress were the best known, with 182 (47.4%) and 178 (46.35%) participants, respectively. Relatively better-known risk factors were diabetes mellitus (138 participants, or 35.94%), hypercholesterolemia (98 participants, or 25.52%), family history (122 participants, or 31.77%), and aging (94 participants, or 24.48%). The poorest knowledge among participants was for risk factors such as obesity (26 participants, or 6.77%) and high salt diet (22 participants, or 5.73%).

**Table 3:** Prevalence of knowledge of risk factors among the study population

Heart attack risk factor	Prevalence of knowledge
Hypertension	182 (47.4%)
Stress	178 (46.35%)
Aging	94 (24.48%)
Family history	122 (31.77%)
Alcoholism	66 (17.19%)
Obesity	26 (6.77%)
Lack of exercise	66 (17.19%)
High salt diet	22 (5.73%)
Smoking	62 (16.14%)
Hypercholesterolemia	98 (25.52%)
Diabetes mellitus	138 (35.94%)

The average scores of the study population are depicted in Table 4. The average risk factor score was 7.61, the average symptom score was 5.56, and the overall heart attack knowledge questionnaire score was 13.18.

**Table 4:** Average scores of the study population

Category	Average score
Risk factor	7.61 (0 - 11)
Symptom	5.56 (0 - 9)
Heart attack knowledge score	13.18 (0 - 20)

## Discussion

The present study identified poor knowledge of heart attack symptoms such as nausea, and neck, back, and epigastric pain. It also highlighted a lack of awareness of risk factors like a high salt diet and obesity. Finally, the study found that the average knowledge score among the population was 13.18 out of 20, confirming our hypothesis. 

In the present study, we explored the awareness of heart attack symptoms and risk factors among the elderly population in rural Chennai, focusing on individuals aged 60 and above. The "golden time" period, during which prompt medical intervention can significantly alter the outcome, underscores the need for greater public awareness and targeted public health strategies, especially in vulnerable age groups [8]. Our findings indicate a considerable gap in knowledge regarding both the symptoms of heart attacks and the associated risk factors within this rural demographic.

Studies conducted in urban settings tend to show a higher level of awareness [9]. This is likely due to better access to healthcare resources and information. However, in rural areas, particularly among the elderly, there is a clear lack of education about cardiovascular health, which may be exacerbated by limited access to healthcare services and a general lack of health literacy [10]. These disparities are concerning, as they may contribute to delayed medical intervention and poorer health outcomes.

Comparing our results with previous studies on similar populations reveals some significant insights [7]. Demographic characteristics showed the education status of the participant population, highlighting the requirement for awareness in this population. Chest pain, breathlessness, light-headedness, and shoulder pain were found to be the best-known symptoms. Awareness was particularly low for epigastric pain, nausea, back pain, and neck pain. Findings were also similar to previous studies, where nearly 44% of respondents had insufficient knowledge, and less than 20% had highly satisfactory knowledge [11]. Hypertension and stress were the best-known risk factors. Studies have also taken this one step further by evaluating and assessing the reasons for the prevalence of these risk factors as well [12]. The poorest knowledge was seen in obesity and a high-salt diet. This was found to be different from previous studies conducted in other rural populations, which reported other risk factors, such as lack of exercise, as poorly known [13].

Additionally, while the elderly in rural Chennai are exposed to common risk factors for heart disease, such as hypertension, diabetes, and smoking, awareness of these risk factors and their implications remains insufficient [14]. This is especially troubling given that many elderly individuals in rural areas may not have regular check-ups or access to preventive health services [15].

Limitations of this study should also be considered. While we obtained a large sample size from the rural field practice area of MMC (Medavakkam), the study was confined to a specific geographic location. Moreover, the study relied on self-reported data, which may carry biases or inaccuracies, particularly when participants have limited health knowledge. Despite these limitations, the present study provides valuable insights into the need for increased awareness and educational interventions targeting the elderly in rural communities.

## Conclusions

This study highlights the crucial gap in awareness regarding MI symptoms and risk factors among the elderly population in rural Chennai. This study uniquely focuses on evaluating both spontaneous and prompted awareness of MI among elderly individuals in a rural South Indian setting. Early recognition of heart attack symptoms and risk factors is vital for timely medical intervention, which can significantly reduce mortality and improve outcomes. Our findings suggest that there is a pressing need for targeted health education programs, particularly in rural areas, to improve the understanding of heart attacks and promote preventive measures.

In conclusion, addressing this knowledge gap through community-based interventions and accessible healthcare services will play a pivotal role in enhancing the health and well-being of the elderly population. These efforts should aim not only to inform but also to empower individuals to seek immediate medical attention, thereby improving overall health outcomes in rural settings.

## Appendices

**Table 5:** Heart attack knowledge questionnaire

Heart attack symptoms	Heart attack risk factors
Chest pain	Hypertension
Breathlessness	Stress
Light-headedness	Aging
Pain in shoulder/arm	Family history
Epigastric pain	Alcoholism
Nausea	Obesity
Cold sweat	Lack of exercise
Back pain	High salt diet
Neck pain	Smoking
	Hypercholesterolemia
	Diabetes mellitus
